# The high sodium condiments and pre-packaged food should be the focus of dietary sodium control in the adult Shanghai population

**DOI:** 10.1186/s12986-022-00692-2

**Published:** 2022-08-25

**Authors:** Zhengyuan Wang, Zhenni Zhu, Hua Cai, Baozhang Luo, Zehuan Shi, Yongping Liu, Xuesong Xiang, Jiajie Zang, Jin Su

**Affiliations:** 1 Division of Health Risk Factors Monitoring and Control, Shanghai Municipal Center for Disease Control and Prevention, 1380# West Zhongshan Road, Changning District, Shanghai, China; 2 School of Public Health, Fudan University, Shanghai, China; 3 Element Nutrition of National Health Commission, National Institute of Nutrition and Health, China Center for Disease Control and Prevention, Beijing, China

**Keywords:** Sodium, Shanghai, Pre-packaged food, High sodium condiments, Seasonality

## Abstract

**Background:**

Long-term, excessively high sodium consumption can lead to increased blood pressure, which is a major risk factor for cardiovascular disease. Therefore, we aimed to analyze the dietary sodium intake and food sources to understand the epidemiological characteristics associated with potentially influencing variables in adults from Shanghai.

**Methods:**

Residents aged 15 years and above were randomly selected using multi-stage stratified random sampling in Shanghai. Over 3 days, family condiments were weighed for each 24-h day, and recall surveys were conducted for the same timeframe regarding sodium intake during the spring, summer, autumn, and winter seasons.

**Results:**

The median sodium intake for residents aged 15 years and above was 4.3 g/d in Shanghai, where 55.1% was obtained from cooking salt, 13.2% from sodium condiments, and 22.2% from pre-packaged food. There were no significant differences in total sodium intake or main sources of sodium intake between different seasons. The sodium intake of rural residents > suburban residents > urban residents (*P* < 0.05). The logistic regression demonstrated that compared to the rural, the people living in urban and suburban consumed less sodium. Compared to the 18–44, the people aged 45–59 and ≥ 60 consumed more sodium (*P* < 0.05).

**Conclusions:**

Sodium intake is high in Shanghai. The absolute amount of cooking salt is low in Shanghai, and the possibility of further reduction is very little under the existing dietary habit. Limiting high sodium condiments and pre-packaged food is the new key to controlling salt intake in the future.

## Background

Sodium is an essential micronutrient and is important in maintaining the body's normal physiological function. However, long-term, excessively high sodium consumption can lead to increased blood pressure, a major risk factor for cardiovascular disease, stroke, and chronic kidney disease [1–4].

Reducing sodium is one of the most cost-effective strategies to prevent chronic diseases, according to the World Health Organization (WHO) [5]. Globally, it has been reported that 4.1 million deaths and 83 million years of disability were attributable to excess dietary sodium intake in 2015 [6]. Data from the 2013 Global Burden of Disease study in China showed that deaths attributable to high-sodium diets accounted for 12.6% of all deaths, 14.5% of chronic disease deaths, and 22,759 million disability-adjusted life years [7]. The WHO global action plan for the prevention and control of non-communicable diseases (2013–2020) stated that there should be a 30% relative reduction in the population’s intake of salt, sodium, or both in persons aged 18 years old and above [8]. Many studies have shown that dietary sources of sodium vary among different regions. Processed foods contribute heavily to sodium intake in the United Kingdom (95%), while the level of sodium in foods consumed outside the home (eating out) contributed to 71% of the dietary salt intake in the United States. Conversely, 76% of dietary sodium was from salt alone added in home cooking in China, and 63% of dietary sodium came from soy sauce, commercially processed fish, seafood, salted soups, and preserved vegetables in a study in Japan [9–11].

To date, no studies have examined the change in trends in the amount and proportion of daily sodium intake derived from salt, monosodium glutamate, soy sauce, and snack food sources in different seasons and regional subgroups. Therefore, we established a study in Shanghai and tracked sodium intake from both dietary and weighing surveys in four seasons from 2012 to 2014. Our study aimed to analyze sodium intake and food sources to understand the epidemiological characteristics of different seasons and regions and whether these were influential factors among Shanghai residents aged 15 and above.

## Methods

### Study population and design

The Shanghai Diet and Health Surveillance (SDHS) is a study that has been conducted since 2012. Four survey waves occurred during 2012–2014. The target population of SDHS is the residents who have lived in Shanghai for more than 6 months over the past year.

**Fig. 1:**
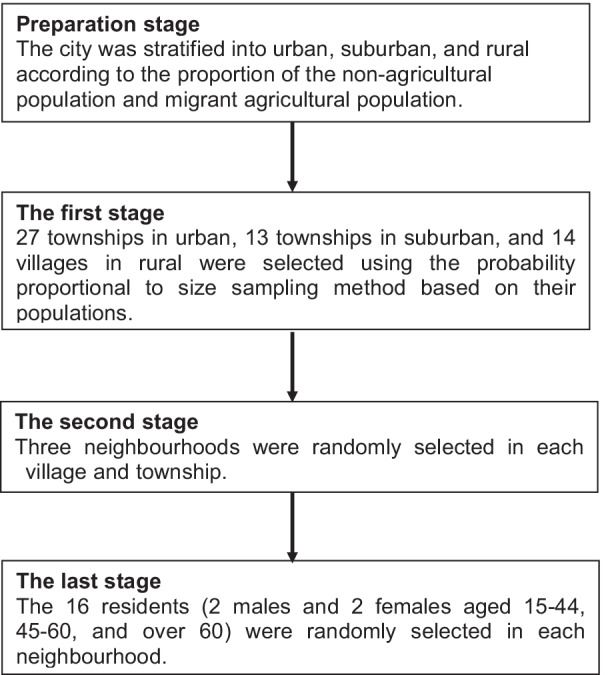
Flow chart of participant recruitment

A multi-stage, stratified random sampling method was used to obtain a representative sample of the Shanghai population aged 15 years and over. The formula for calculating stratified random sampling sample size, n = z^2^*S^2^*deff/d^2^, was used to calculate the sample size required for analysis. We defined the two-sided significance levels α = 0.05, 1 − β = 0.8, and z_α/2_ = 1.96. The deff value of stratified random sampling was 1. According to the variation in sodium intake, we needed at least 1496 subjects. Considering participants’ refusal and the loss to follow-up, we recruited a total of 1944 subjects aged 15 years old and above into the study. The city was stratified into urban, suburban, and rural. The sampling number of each layer for urban, suburban, and rural was 27, 13, and 14 respectively, according to the total population of each layer. Villages and townships within the urban, suburban, and rural areas were selected using the probability-proportional-to-size sampling method based on their populations. Three neighborhoods were randomly selected in each village and township. The residents were randomly selected in each neighborhood, including three age groups (15–44 years, 45–59 years, and over 60 years old), and each group comprised two males and two females. The detailed methods have been described previously [12] (Fig. 1).

### Dietary survey

Every subject was asked to recall all food consumed over the previous 24 h for 3 days (two working days and one weekend day) and the place where the food was consumed to collect general food intake data. A household condiment weighing method was implemented to collect condiment data (such as edible oils, table salt, monosodium glutamate, and soy sauces). It was determined by weighing changes in the condiment inventory from the beginning to the end of each day. All purchases and wasted condiments were also weighed. At the same time, the basic health characteristics of people who consumed the household condiments at each meal, including family members and guests, were recorded. Characteristics included gender, age, and physical condition. Three-day 24-h recall was performed on three consecutive days to match with the weighing of the condiments. Participants were required to complete the standardized questionnaire face-to-face with trained interviewers. The interviewers went to each subject’s house and recorded the types and amounts of food consumed by subjects using food picture aids and the questionnaire. Household condiments were weighed during the previous day and every night after dinner by trained interviewers with the same brand and model of scales, four nights per participant. Individual condiment consumption was calculated according to the total amount of condiment consumed in the household divided by the proportions of energy consumption of individuals in the household. The sodium from eating out condiments was estimated and converted according to eating out energy intake based on energy and condiment sodium intake ratio on home eating. All data were reviewed by the local district Centers for Disease Control and Prevention (CDC) project team, and then at least 5% of data were reviewed by the Shanghai municipal CDC project team. If the quality control result was “disqualified” from Shanghai municipal CDC project team, all data should be re-checked by the local district CDC project team. Individual condiment consumption was calculated according to the total amount of condiment consumed in the household divided by the proportion of energy consumption of individuals and eating conditions in the household, which has been described previously [12]. The amount of sodium in food was based on the Chinese food composition table, the most authoritative and complete tool for food ingredients in China [13]. The amount of sodium in pre-packaged foods was based on the self-made pre-packaged food composition table according to the nutrition information on the food bags. Total sodium intake was equal to the sum of sodium from cooking salt, monosodium glutamate, soy sauce, pre-packaged foods, and raw foods. The cooking salt, monosodium glutamate, say sauce, and pre-packaged food were the main sources of sodium intake and can be controlled to reduce sodium intake.

### Physical examination

The physical examination was conducted only once during the first investigation. A total of 98.6% of subjects completed a physical examination during the spring survey. Physical examination was implemented directly by trained health workers, using a standard protocol. The TZG height measurement was used to assess height, the SECA882 Electron weight scale was used for weight, and the Graham-Field 1340–2 was used to measure waist circumference and hip circumference. All physical examination equipment was certified and qualified before use.

### The definition

Lastly, season was defined as spring, summer, fall (autumn), and winter according to the time of the survey. Pre-packaged foods refer to the processed foods pre-packaged or prepared in packaging materials and containers. Body mass index (BMI) was divided into four categorical levels based on the criteria recommended by the National Health and Family Planning Commission of the People’s Republic of China. These are underweight (BMI: < 18.5 kg/m^2^), normal (BMI: 18.5–23.9 kg/m^2^), overweight (BMI: 24.0–27.9 kg/m^2^), and obese (BMI: ≥ 28.0 kg/m^2^).

### Statistical analysis

Weighted statistical analysis based on the complex sampling design was used for all measurements. The weighting was based on data from the sixth national census in 2010. Missing data and data where the daily energy intake for a standard person was less than 2094.9 kJ (500 kcal) or exceeded 20,910 kJ (5000 kcal) were removed. We considered the following confounders and adjusted for these: Weights of sampling design, age, gender stratification, and lack of answers. Mean and standard deviation (SD) were used to evaluate normally distributed data, while non-normally distributed data were evaluated by the median, 25th percentile (P25), and 75th percentile (P75). A generalized estimation model was used to analyze the comparison among different seasons. This analysis included only subjects who completed the survey four times. Kruskal Wallis-one-way ANOVA test (K) was used to compare the non-parametric data among different regions. Pairwise comparisons were conducted by the pairwise method. The multivariable logistic regression (forward stepwise) was used for univariate analyses. The criterion for inclusion in the regression model was 0.05, and the criterion for exclusion was 0.1. Statistical significance was set at *P* < 0.05. Coefficient and 95% confidence intervals were calculated. The data for average sodium intake across the four seasons was used in both univariate and multivariate linear regression models. All statistical analyses were performed using the statistical/data analysis software package SAS 9.3 (SAS, Cary, NC, USA).

## Results

### Characteristics of participants

A total of 178 respondents were deleted, and 1524 subjects fully participated in the surveillance. Overall, 1339 subjects completed the survey four times, 116 completed it three times, 45 completed it twice, and 24 individuals completed it once. The proportions of men and women were 49.5% and 50.5%, respectively. The mean age was 54.4 ± 16.9 years (Table 1). The mean energy intake, weight, BMI, and waist circumference were 8571 kJ, 62.5 kg, 23.5 kg/m^2^, and 82.3 cm.

**Table 1:** Demographics of the surveillance subjects

Season	Sample size (person-time)				Gender (n)		Age (years) (mean ± SD)
	Total	Urban	Suburban	Rural	Male	Female	
Spring	1503	703	367	433	750	753	54.6 ± 17.0
Summer	1448	669	361	418	710	738	54.6 ± 16.9
Autumn	1444	666	359	419	722	722	54.0 ± 16.9
Winter	1423	658	352	413	700	723	54.5 ± 16.8
All	5818	2696	1439	1683	2882	2936	54.4 ± 16.9

### Sodium intake and food sources

The median sodium intake for residents aged 15 and above was 4306 mg/d in Shanghai, including 2373 mg (55.1%) from cooking salt, 131 mg (3.0%) from monosodium glutamate, 439 mg (10.2%) from soy sauce, and 479 mg (22.2%) from pre-packaged food (Table 2).

**Table 2:** Intake of salt monosodium glutamate and sauce in different seasons and regions in Shanghai (per standard person per day) (median (P25, P75)) (mg)

Region	Season	n	Sodium	Source of sodium			
				Cooking salt	Monosodium glutamate	Soy sauce	Pre-packaged food
Shanghai	Spring	1503	4167 (3017.6232)	2369 (1455.3741)	139 (42.281)	451 (160.876)	858 (114.3232)
	Summer	1448	4194 (2915.6078)	2366 (1485.3691)	122 (30.262)	407 (167.857)	1216 (112.3536)
	Autumn	1444	4288 (2968.6029)	2354 (1454.3693)	131 (30.264)	440 (208.919)	1136 (104.4004)
	Winter	1423	4515 (3037.6432)	2452 (1432.3866)	132 (31.274)	451 (169.833)	784 (92.3174)
	On average	1524	4306 (2973.6202)	2373 (1451.3744)	131 (34.274)	439 (171.870)	958 (106.3452)
Urban	Spring	703	4129 (2935.6232)^a^	2341 (1455.3673)^a^	132 (39.257)^a^	415 (148.844)^a^	1236 (158.3594)^a^
	Summer	669	4132 (2855.5697)	2353 (1481.3639)	112 (13.237)^a^	379 (155.797)^a^	1782 (212.3866)^a^
	Autumn	666	4284 (2917.5938)^a^	2329 (1415.3621)^a^	105 (0.238)^a^	417 (193.821)^a^	2058 (176.4788)^a^
	Winter	658	4476 (2953.6280)^a^	2487 (1405.3840)^a^	134 (19.245)^a^	400 (153.760)^a^	1094 (128.3594)^a^
	On average	713	4247 (2921.6087)^a^	2366 (1436.3673)^a^	122 (23.245)^a^	407 (161.803)^a^	1414 (160.3866)^a^
Suburban	Spring	367	4195 (3321.5837)	2363 (1325.3641)	191 (72.315)	530 (229.966)	344 (66.3032)
	Summer	361	4440 (3232.7450)	2431 (1485.4954)	142 (61.339)	434 (223.950)	386 (52.3184)
	Autumn	359	4135 (3122.5827)	2134 (1442.3734)	192 (82.335)	434 (228.1113)	254 (68.2584)
	Winter	352	4453 (3227.6432)	2233 (1368.3741)	144 (67.313)	483 (227.1009)	448 (66.2786)
	On average	372	4350 (3246.6272)	2327 (1381.3776)	176 (70.318)	471 (228.1031)	370 (64.2934)
Rural	Spring	433	4362 (3254.6373)	2641 (1692.4415)	126 (62.284)	636 (182.1036)	150 (0.974)
	Summer	418	4352 (2828.6662)	2355 (1497.3839)	144 (65.298)	569 (239.1233)	102 (0.534)
	Autumn	419	4646 (3326.6700)	2705 (1738.4350)	167 (72.370)	644 (248.1221)	120 (0.1146)
	Winter	413	4811 (3199.7089)	2591 (1694.4424)	121 (36.321)	684 (228.1270)	160 (0.1214)
	On average	439	4516 (3146.6712)	2591 (1677.4284)	136 (57.321)	636 (221.1204)	126 (0.974)

^a^The analyses were conducted in different regions within the same season (*P* < 0.05)

According to the application requirements of the generalized estimation model, only 1339 subjects who completed the survey four times were analyzed in the comparison of differences among the four seasons, 626 in urban, 331 in suburban, and 382 rural. There were no significant differences in total sodium intake or the four main sources of sodium intake between the different seasons.

Regarding the intake of sodium, there were statistically significant differences among the different regions (*P* < 0.05) except for intake of total sodium and sodium for cooking salt in summer, which were not significantly different. Over the study period, there was a trend in the total sodium intake and sodium from salt and soy sauce, where the intake of rural residents > suburban residents > urban residents.

### Factors associated with total sodium intake

The data for average sodium intake across the four seasons was used in both univariate and multivariate linear regression models. According to the P25 of average sodium intake, the subjects were divided into two groups: the low sodium intake group (< 2973 mg/d) and the high sodium intake group (≥ 2973 mg/d). Multivariable logistic regression analyses were conducted using gender, region, age, energy intake, and BMI as independent variables and average sodium intake as the dependent variable (Table 3).

**Table 3:** Factors associated with high sodium intake

Items	N	β	Coeff	95% CI	*P*
*Gender*					
Female	765	Reference			
Male	759	2.625	/	/	0.105
*Region*					
Rural	436	Reference			
Urban	714	− 0.710	0.492	0.348–0.694	< 0.001
Suburban	374	− 0.754	0.470	0.323–0.685	< 0.001
*Age (years)*					
18–44	418	Reference			
45–59	506	0.355	1.427	1.006–2.024	0.046
≥ 60	600	0.498	1.645	1.169–2.317	0.004
*Energy*					
< EER in China	878	Reference			
≥ EER in China	646	1.375	3.955	2.981–5.247	< 0.001
*BMI*					
Under weight	89	Reference			
Normal	769	0.527	1.695	1.052–2.729	0.030
Overweight	498	0.647	1.963	1.189–3.241	0.008
Obese	168	0.899	2.457	1.346–4.483	0.003

*EER* Estimated energy requirement

Compared to the people living in rural, the people living in urban and suburban consumed less sodium (coefficient = 0.492, 95% CI 0.348–0.694, *P* < 0.001; coefficient = 0.470, 95% CI 0.323–0.685, *P* < 0.001). Compared to the people aged 18–44, the people aged 45–59 and ≥ 60 consumed more sodium (coefficient = 1.427, 95% CI 1.006–2.024, *P* = 0.046; coefficient = 1.645, 95% CI 1.169–2.317, *P* = 0.004). No significant differences were observed according to gender.

## Discussion

Excessive sodium intake was a major risk factor for cardiovascular disease, stroke, and chronic kidney disease, and other diseases. The dangers of excessive sodium intake have drawn global attention. Many studies have reported that elevated sodium intake is associated with a number of non-communicable chronic diseases (including hypertension, cardiovascular disease, and stroke) and that a decrease in sodium intake may reduce blood pressure and the risk of these associated non-communicable chronic diseases [14–16]. Indeed, the WHO showed that reducing sodium intake could significantly reduce blood pressure in all populations and that reducing sodium intake to < 2000 mg/d was more beneficial than reducing sodium intake but still consuming > 2000 mg /d. However, the WHO also reported no association between sodium intake and all-cause mortality, including cardiovascular disease and non-fatal coronary heart disease [17]. A British study has shown that a high-salt diet can lead to obesity, independent of energy or sugar intake. The risk of childhood and adult obesity increased by 28% and 26% with an increased salt intake of 1 g/d [18]. Furthermore, a meta-analysis of 18 cross-sectional studies showed that the higher a person’s sodium intake, the larger their waistline [19].

This study evaluated sodium intake status and associated epidemiological factors for Shanghai residents enrolled in the SDHS from 2012 to 2014 across four seasons in each calendar year. The results showed that the median sodium intake for residents aged 15 years old and above was 4306 mg/d in Shanghai, and the salt intake from cooking salt was just 6.0 g/d in our study. A study of 6072 adults from 12 mainland provinces from 2009 to 2011 found that the intake of cooking salt was 9.2 g/d in China as a whole but only 6.7 g/d in Shanghai [20]. The difference may be related to the lower intake of cooking salt in Shanghai, which may be related to a series of salt-reduction health education, including the city government distributing free salt control spoons to all families [21]. Moreover, we found that the total sodium intake and main food sodium intake source did not alter significantly with the changing seasons. This phenomenon may be related to the convenience of shopping for these foods.

The average sodium intake of Shanghai residents aged 15 years and over was 4915 mg/d, representing a 13.1% decline from the national level of 5702 mg/d that that was reported in a Chinese nutrition and health monitoring study from 2010 to 2013. Furthermore, this was well below the previous average of a national nutrition survey (6268 mg/d in 2002, a 9.0% decline) [22]. This finding may be linked with the salt control program [21], increased publicity of the program in mainstream media, and the free distribution of salt control scoops. All these factors would encourage Shanghai residents to consciously focus on their daily salt consumption, guiding the formation of improved health behaviors. However, although the total sodium intake in Shanghai is far below the national average, unfortunately, it is still above the global mean sodium intake (3900 mg/d) [23]. Furthermore, it far exceeds the WHO recommended standard, which states that the adult’s daily sodium intake should be less than 2000 mg [17]. The Chinese dietary reference intake standard states that the recommended nutrient intake of sodium should be 1500 mg/d in people aged 18 years old and over because it is likely that non-communicable chronic disease can be prevented if the intake does not exceed 2000 mg/d [24]. In our study, the sodium intake was approximately 2.5 times the recommended standard. From an individual perspective, 96.4% of Shanghai residents had a higher sodium intake than the 2000 mg/d level.

The essence of controlling salt is sodium restriction; the consumption of visible salt (cooking salt) is already low in Shanghai, and we found that it approximated 55.1% of the total sodium intake. The next step in controlling salt should be targeting salt-containing condiments, such as monosodium glutamate, soy sauce, and pre-packaged foods. The Chinese National Centre for Food Safety Risk Assessment showed that sodium intake from non-cooking salt increased by 12.6% from 2009 to 2011[19]. In our study, the sodium from monosodium glutamate, soy sauce, and pre-packaged food approximated 35.4% of the total sodium intake. In the last 20 years, with the gradual development of food sales from traditional single outlets to modern food chain supermarkets, convenience stores, and online shopping systems, residents select and purchase more pre-packaged food. Consequently, global pre-packaged food consumption is increasing [25]. Some studies have shown that the pre-packaged food consumption rate of the adult population in China is 85.3%. Among these pre-packaged foods, the sodium content of convenience foods and baked goods is generally high, and the consumption rate is also high, at 52.8% and 31.7%, respectively [26]. Our study found that Shanghai residents consumed approximately 22.2% of sodium from pre-packaged foods. We believe that this has been substantially undervalued. Chinese dietary composition tables contained approximately 300 kinds of pre-packaged foods, and these were all domestic foods. As an international metropolis, people in Shanghai can easily buy all pre-packaged foods, including imported food, which can now be included as part of the Chinese dietary composition in Shanghai. When we processed the data, we used a similar pre-packaged food or the main raw material of the pre-packaged food to replace the pre-packaged food that was not in the database. When we substituted pre-packaged foods for the main raw material of the pre-packaged food, the salt added to the pre-packaged foods during processing was ignored. An Australian study, which assessed the sodium content of 15,680 pre-packaged foods in the supermarket, found that the average sodium content was 500 mg/100 g of food in Australia. Voluntary salt control in the food industry is the most economical and effective salt reduction policy [6]. People can adapt to the taste of processed food with its salt intake reduced by 10–20 percent, which does not affect consumption [27].

Nutritional labeling is one of the most recognized and effective nutrition interventions worldwide. Labeling regulations have been adopted in many countries experiencing a nutrition transition from traditional diets to contemporary patterns of food consumption [28]. Labeling regulations have been initiated in Europe, North America, Australia, New Zealand, Asia, Africa, the Middle East, and Latin America [29]. In 2011, China enacted the General Rules for Nutrition Labeling of Pre-packaged Foods (GB 7718-2011), the first mandatory legislation on nutritional labeling in China. The general rules stipulated that all pre-packaged foods must label the contents of energy, protein, fat, carbohydrate, and sodium in a prominent and easily viewed place. The labeling rate for the sodium content of pre-packaged foods was 99.8% in Shanghai [30]. From a public health perspective, it is important to teach residents to recognize and use nutrition labels to choose low-sodium foods and discourage people from consuming too much sodium in pre-packaged foods.

The limitation of the present study is that the study design may have underestimated the surplus of cooking salt and high sodium condiments. We used the proportion of condiment intake at home to estimate the intake of condiments eaten out, accordingly. However, we found that Shanghai residents have tended to dine out more often in recent years because of the development of the economy and the online takeaway industry. In 2016, China's online food takeaway turnover was about 17.0 billion USD. In general, food consumed while eating out tends to have higher levels of salt than homemade food [31]. We also recognize that caution should be taken when generalizing our results to the global population, as differences in food preferences, tastes, cooking habits, lifestyles, and health consciousness might exist between the general population and our study population.

## Conclusions

The total sodium intake of residents aged 15 years and above is lower than the national average level but still very high in Shanghai, which may increase the prevalence of non-communicable chronic diseases such as hypertension. Although the proportion of sodium from cooking salt is 55.1%, the absolute amount of cooking salt is very close to the standard recommended by WHO, and the possibility of further reduction is very little. The focus of sodium control in Shanghai should be transferred from cooking salt to high sodium condiments and pre-packaged foods, especially salt control within the food industry. Our findings suggested that the further away from the city center, the greater the participant’s energy intake, which could be considered a risk factor that might have affected the sodium intake.

## Data Availability

Details of how to access the data are available from the first author and correspondence authors.

## Abbreviations

WHO: World Health Organization
SDHS: Shanghai Diet and Health Surveillance
CDC: Centers for Disease Control and Prevention (which is the government agency in China)
SD: Standard deviation

## References

[1] Farquhar WB, Edwards DG, Jurkovitz CT (2015). Dietary sodium and health: more than just blood pressure. J Am Coll Cardiol.

[2] Aburto NJ, Ziolkovska A, Hooper L (2013). Effect of lower sodium intake on health: systematic review and meta-analyses. BMJ.

[3] He FJ, Li J, Macgregor GA (2013). Effect of longer-term modest salt reduction on blood pressure: cochrane systematic review and meta-analysis of randomised trials. Cochrane Database Syst Rev.

[4] Mozaffarian D, Singh GM, Powles J (2014). Sodium and cardiovascular disease: what the data show. New Engl J Med.

[5] Hanson M, Gluckman P, Nutbeam D (2011). Priority actions for the non-communicable disease crisis. Lancet.

[6] Collaborators GBDRF (2016). Global, regional, and national comparative risk assessment of 79 behavioural, environmental and occupational, and metabolic risks or clusters of risks, 1990–2015: a systematic analysis for the Global Burden of Disease Study 2015. Lancet.

[7] Liu M, Li YC, Liu SW (2016). Burden of disease attributable to high-sodium diets in China, 2013. Chin J Prev Med.

[8] WHO (2015). Global action plan for the prevention and control of noncommunicable diseases (2013–2020).

[9] Anderson CA, Appel LJ, Okuda N (2010). Dietary sources of sodium in China, Japan, the United Kingdom, and the United States, women and men aged 40 to 59 years: the INTERMAP study. J Am Diet Assoc.

[10] Shao S, Hua Y, Yang Y (2017). Salt reduction in China: a state-of-the-art review. Risk Manag Healthc Policy.

[11] Harnack LJ, Cogswell ME, Shikany JM (2017). Sources of sodium in US adults from 3 geographic regions. Circulation.

[12] Zang JJ, Yu HT, Zhu ZN (2017). Does the dietary pattern of shanghai residents change across seasons and area of residence: assessing dietary quality using the Chinese diet balance index (DBI). Nutrients.

[13] Yang Y (2002). China food composition.

[14] Bibbins-Domingo K, Chertow GM, Coxson PG (2010). Projected effect of dietary salt reductions on future cardiovascular disease. N Engl J Med.

[15] Strazzullo P, D'Elia L, Kandala NB (2009). Salt intake, stroke, and cardiovascular disease: meta-analysis of prospective studies. BMJ.

[16] He FJ, MacGregor GA (2009). A comprehensive review on salt and health and current experience of worldwide salt reduction programmes. J Hum Hypertens.

[17] WHO (2012). Sodium intake for adults and children guideline.

[18] Ma Y, He FJ, MacGregor GA (2015). High salt intake: Independent risk factor for obesity?. Hypertension.

[19] Moosavian SP, Haghighatdoost F, Surkan PJ (2017). Salt and obesity: a systematic review and meta-analysis of observational studies. Int J Food Sci Nutr.

[20] Hipgrave DB, Chang S, Li X (2016). Salt and sodium intake in China. JAMA.

[21] Wang Y, Shi Y, Wu C (2015). Evaluation on a mass campaign for salt control in Shanghai: change in salt consumption. J Environ Occup Med.

[22] Jile C, Yu W (2016). Chinese nutrition and health monitoring (2010–2013 year comprehensive report).

[23] Powles J, Fahimi S, Micha R (2013). Global, regional and national sodium intakes in 1990 and 2010: a systematic analysis of 24 h urinary sodium excretion and dietary surveys worldwide. BMJ Open.

[24] Chinese Society of Nutrition (2014). Chinese dietary reference intakes.

[25] Ng SW, Dunford E (2013). Complexities and opportunities in monitoring and evaluating US and global changes by the food industry. Obes Rev.

[26] Huang F, Zhang J, Wang H (2015). Pre-packaged foods' nutritional ingredients analysis among 706 adult residents in cities in China. Chin J Prev Med.

[27] Brinsden HC, He FJ, Jenner KH, Macgregor GA (2013). Surveys of the salt content in UK bread: progress made and further reductions possible. BMJ Open.

[28] Nazmi A, Monteiro C (2013). The nutrition transition: the same, but different. Public Health Nutr.

[29] Mandle J, Tugendhaft A, Michalow J (2015). Nutrition labelling: a review of research on consumer and industry response in the global South. Glob Health Action.

[30] Kong K, Liu F, Tao Y (2017). The presence and accuracy of nutritional labelling of pre-packaged foods in Shanghai. Asia-Pac J Clin Nutr.

[31] Byrd K, Almanza B, Ghiselli RF (2017). Reported action to decrease sodium intake is associated with dining out frequency and use of menu nutrition information among US adults. J Acad Nutr Diet.

